# Genetic Dissection of Novel QTLs for Resistance to Leaf Spots and Tomato Spotted Wilt Virus in Peanut (*Arachis hypogaea* L.)

**DOI:** 10.3389/fpls.2017.00025

**Published:** 2017-01-31

**Authors:** Manish K. Pandey, Hui Wang, Pawan Khera, Manish K. Vishwakarma, Sandip M. Kale, Albert K. Culbreath, C. Corley Holbrook, Xingjun Wang, Rajeev K. Varshney, Baozhu Guo

**Affiliations:** ^1^Crop Protection and Management Research Unit, United States Department of Agriculture, Agricultural Research ServiceTifton, GA, USA; ^2^International Crops Research Institute for the Semi-Arid TropicsHyderabad, India; ^3^Department of Plant Pathology, University of GeorgiaTifton, GA, USA; ^4^Crop Genetics and Breeding Research Unit, United States Department of Agriculture, Agricultural Research ServiceTifton, GA, USA; ^5^Biotechnology Research Center, Shandong Academy of Agricultural SciencesJinan, China

**Keywords:** tomato spotted wilt virus (TSWV), early leaf spot (ELS), late leaf spot (LLS), quantitative trait locus (QTL), peanuts

## Abstract

Peanut is an important crop, economically and nutritiously, but high production cost is a serious challenge to peanut farmers as exemplified by chemical spray to control foliar diseases such as leaf spots and thrips, the vectors of tomato spotted wilt virus (TSWV). The objective of this research was to map the quantitative trait loci (QTLs) for resistance to leaf spots and TSWV in one recombinant inbred line (RIL) mapping population of “Tifrunner × GT-C20” for identification of linked markers for marker-assisted breeding. Here, we report the improved genetic linkage map with 418 marker loci with a marker density of 5.3 cM/loci and QTLs associated with multi-year (2010–2013) field phenotypes of foliar disease traits, including early leaf spot (ELS), late leaf spot (LLS), and TSWV. A total of 42 QTLs were identified with phenotypic variation explained (PVE) from 6.36 to 15.6%. There were nine QTLs for resistance to ELS, 22 QTLs for LLS, and 11 QTLs for TSWV, including six, five, and one major QTLs with PVE higher than 10% for resistance to each disease, respectively. Of the total 42 QTLs, 34 were mapped on the A sub-genome and eight mapped on the B sub-genome suggesting that the A sub-genome harbors more resistance genes than the B sub-genome. This genetic linkage map was also compared with two diploid peanut physical maps, and the overall co-linearity was 48.4% with an average co-linearity of 51.7% for the A sub-genome and 46.4% for the B sub-genome. The identified QTLs associated markers and potential candidate genes will be studied further for possible application in molecular breeding in peanut genetic improvement for disease resistance.

## Introduction

Peanut (*Arachis hypogaea* L.) is an economically-important legume, and a major source of protein (25–28%) and vegetable oil (43–55%) for human nutrition. The peanut production in India and China together accounts for almost two thirds of the world's peanuts, and the U.S. produces about 6% (Guo et al., 2012). Two thirds of peanut production is crushed for oil, and one third is consumed as food. High production cost is a challenge to peanut growers because of chemical spray to control diseases. Early leaf spot (ELS) caused by *Cercospora arachidicola* and late leaf spot (LLS) caused by *Cercosporidium personatum* are two important diseases worldwide (Backman and Crawford, 1984). Epidemics of leaf spot diseases can cause complete defoliation, resulting in significant yield losses. Epidemics are affected by weather patterns such as hot and wet conditions (Shew et al., 1988). Control of leaf spot in the U.S. depends on scheduled applications of fungicide (Culbreath et al., 2002). In the U.S., tomato spotted wilt virus (TSWV) has been an important factor of concern for breeders and farmers, and the control methods are limited (Culbreath et al., 2003; Culbreath and Srinivasan, 2011). TSWV is primarily transmitted by thrips, *Frankliniella fusca* (tobacco thrips), and *F. occidentalis* (western flower thrips) in the U.S. From 1996 to 2006, TSWV disease alone caused the annual losses of $12.3 million (Riley et al., 2011).

Conventional breeding has been the major avenue for providing modern peanut cultivars to farmers. Integration of molecular breeding with conventional methods has been successful in some crops but peanut has lagged behind due to lack of molecular markers linked to traits of interest. However, progress in recent years has made it possible to use marker-assisted selection (MAS) in peanut breeding (Varshney, 2016). Informative markers linked to desired traits were deployed in molecular breeding in peanuts successfully such as the peanut cultivar “Tifguard” was converted into “high oleic Tifguard” (Holbrook et al., 2008; Chu et al., 2011) and the multiple elite varieties were improved for rust resistance (Varshney et al., 2014) and oil quality (Janila et al., 2016). Although, molecular breeding has been applied on a limited scale (Pandey et al., 2012a; Guo et al., 2013) for few traits of interest, peanut still lacks availability of linked markers for many important traits including disease resistances.

For peanut research, because the disease pressure varies year to year, multi-year field screening for resistance to diseases is very essential and important (Culbreath et al., 2002, 2005; Culbreath and Srinivasan, 2011). Much of the yearly variation such as leaf spot pressure can be explained by differences in the amount of rainfall during these years. Little is known about the causes of annual variation in severity of TSWV epidemics. In Georgia, all three diseases co-exist, and one disease is predominant than the other, particularly ELS and LLS. For example, in our study, if ELS was predominant we evaluated this disease as ELS or *vice versa*. The search efforts for resistance to TSWV have been intensive in the last decade leading to identification of several sources of resistance in peanut (Culbreath and Srinivasan, 2011; Khera et al., 2016). Peanut with field resistance is characterized as reduced number of diseased plants with typical symptom and higher yields in comparison with susceptible peanut varieties in the field trials (Culbreath et al., 2003). Culbreath and Srinivasan (2011) reported that the observed low incidence of spotted wilt in peanut TSWV-resistant genotypes is not believed to be due to resistance against the vector but due to field resistance against the virus.

Identification of molecular markers is needed to expand application of MAS in peanut for other disease resistances (Varshney et al., 2013). Two major QTLs for LLS resistance and one major QTL for rust resistance were identified using a recombinant inbred line (RIL) population derived from the cross TAG 24 × GPBD 4 (Khedikar et al., 2010; Sujay et al., 2012). Linked SSR markers were validated and deployed through MABC to improve resistance for rust and LLS (Varshney et al., 2014). One major QTL on linkage group AhXV for both rust and LLS and the second major QTL for LLS resistance located on linkage group AhXII (Khedikar et al., 2010; Sujay et al., 2012) have been reassigned to A03 and A02, respectively, after the diploid genome sequences were published (Bertioli et al., 2016). Khera et al. (2016) reported major QTLs with over 10% PVE from parental line “NC94022” (Culbreath et al., 2005) for resistance to TSWV, ELS and LLS, which were assigned primarily on linkage groups A01 (TSWV), A01 and A03 (ELS) and B03 (LLS) (Khera et al., 2016).

Nevertheless, the mapping population (recombinant inbred lines, RILs) derived from the cross “Tifrunner” × “GT-C20” was developed and used for identification of linked markers for leaf spots and TSWV resistance. The parental genotypes have several contrasting traits. Tifrunner has high level of resistance to TSWV, and moderate resistance to early and late leaf spot (Holbrook and Culbreath, 2007) while GT-C20 is very susceptible to these diseases but had reduced levels of aflatoxin contamination (Liang et al., 2005). This population has been used for genetic linkage map construction at F_2_ generation with 318 mapped loci using a set of 94 F_2_ lines (Wang et al., 2012), F_5_ generation with 239 mapped loci (Qin et al., 2012), and F_8:9_ generation as RILs with 378 mapped loci (Pandey et al., 2014). In this study the genetic map was improved by screening more SSR markers for polymorphism, including 199 highly informative genic and genomic SSR markers published by Pandey et al. (2012b), 78 highly polymorphic long TC repeat SSRs developed by Macedo et al. (2012), and 28 SSRs identified from peanut expressed sequence tags (ESTs) derived resistance gene analogs (RGAs) (Liu et al., 2013). In total, 45 polymorphic markers were identified and 40 marker loci were successfully integrated into the existing genetic map (Qin et al., 2012; Pandey et al., 2014), along with the extensive phenotypic data in the field for disease resistances, this study reports the improved genetic map and the identification of QTLs linked to the resistance to ELS, LLS, and TSWV. This map along with the markers and identified QTLs were also compared with the diploid peanut physical maps (Bertioli et al., 2016), serving as a bridge between molecular breeding, map-based cloning and whole genome sequence assembly.

## Materials and methods

### Plant material

A recombinant inbred line (RIL) population was derived from the cross of Tifrunner × GT-C20 (referred as “T-population”) with a population size of 248 (Wang et al., 2013) using single seed decent (SSD) method at the Crop Protection and Management Research Unit, USDA-ARS, Tifton, GA. The female parent Tifrunner is a runner market-type with high level of resistance to TSWV, moderate resistance to ELS and LLS, and late maturity (Culbreath et al., 2005; Holbrook and Culbreath, 2007). GT-C20 is a Spanish-type breeding line with high susceptibility to TSWV and leaf spots (LS). Multi-season phenotyping data were recorded for LS and TSWV traits.

### Field screening and evaluation of disease resistance

The RIL population was evaluated in field trials for leaf spots (LS) including early leaf spot (ELS) and late leaf spot (LLS) and TSWV from 2010 to 2013 at the Bellflower Farm, Tifton, GA, using a randomized complete block design with three replications. It was planted twice each year in the months of April and May. The soil type of experimental site was Tifton loamy sand. The phenotyping data was collected from the field experiment where each RIL was planted in a two-row plot. The length of the each experimental plot was 3.0 m separated by an alley of 1.5 m. The seeding rate was 10 seeds per m. The disease reactions in the field trials were from nature infection. The early planted trials of April were mainly used for the spotted wilt rating in order to increase TSWV pressure, and the late planting was to reduce the TSWV interference with leaf spot rating but both plantings were used for leaf spots (ELS and LLS) evaluation in order to have optimized disease ratings (Li et al., 2012).

During the trial period, to ensure the disease severity for all the three diseases (ELS, LLS, and TSWV), disease scoring was recorded at different dates. Readings (r) were assigned on the basis of the month when the observation was recorded, for example r1 for July, r2 for August and r3 for September. There were a total of 23 successful readings for these diseases from 2010 to 2013. The disease score for LS (ELS and/or LLS) was recorded using the Florida scale 1–10 as described by Chiteka et al. (1988) and Wang et al. (2013). For TSWV, the score of disease incidence was recorded based on 0–5 disease severity scale as described by Baldessari (2008).

### DNA isolation, polymorphism, and genotyping

The DNA was isolated from the young leaflets of all the RILs along with the parental lines (Tifrunner and GT-C20) using the method described in Qin et al. (2012). The genomic DNA was further quantified in Nano Drop-1000 spectrophotometer for quality and quantity. Polymerase chain reactions (PCR) were carried out using good quality DNA in a 25 μl reaction mixture using thermal cycler DNA Engine Tetrad 2 Peltier and PTC-225 DNA Engine Tetrad Peltier. The master mix for PCR was prepared using 25 ng template DNA, 0.5 μM of each primer, 10X PCR buffer, 1.5 mM MgCl, 0.2 mM of dNTPs, and 0.5 U of *Taq* polymerase. Detailed PCR reactions and scoring of PCR bands was followed as described by Qin et al. (2012) and Fountain et al. (2011). In order to improve the map density, we collected newly developed and highly informative SSRs (Macedo et al., 2012; Pandey et al., 2012a; Liu et al., 2013) for screening for polymorphic markers. More markers were used by following the protocol and genotypic method for constructing an improved genetic map based on Qin et al. (2012) and Pandey et al. (2014).

### Genetic linkage map improvement

In 2012, Qin et al. published the first version of the genetic map using this population with 239 marker loci (Qin et al., 2012). Pandey et al. (2014) improved this map to 378 mapped loci. In this study, there were 400 more markers generated for screening the parental lines in order to improve the genetic linkage map further, resulting in 45 newly identified polymorphic markers for the T-population. JoinMap® version 4 (Van Ooijen, 2006) was used for construction of the improved genetic map. Initially, chi-square values were analyzed for these 45 markers to check the segregation distortion using the option “locus genotype frequency” function in the JoinMap. These marker loci were mapped on the existing linkage groups of the earlier genetic map (Pandey et al., 2014) and conversion of the recombination fraction into map distances in centiMorgans (cM) was done using the Kosambi map function (Kosambi, 1944). At recombination frequency of 45%, most of the markers were integrated into the earlier genetic map. Mapchart 2.2 (Voorrips, 2002) was used to visualize final marker positions of each linkage group (LG).

### Comparison with the physical map position of markers in the sub-genomes

The reference genome sequences of two peanut diploid progenitors, *A. duranensis* (A genome, 2*n* = 2*x* = 20) and *A. ipaensis* (B genome, 2*n* = 2*x* = 20), have been completed (www.peanutbase.org; Bertioli et al., 2016). In order to determine the physical positions of these markers on the LG of the linkage map, the sequence of each marker was aligned against the reference genome sequences using BLASTN program and the coordinates of top blast hit for each SSR marker sequence were used to predict the location of the respective marker on peanut sub-genomes. Initially, the BLASTN search was carried out at higher stringency (*e*-value 10^−25^) in order to get highly confident hits. For sequences which did not have any hit, the BLASTN program was run with less stringency (*e*-value 10^−15^) and physical positions were determined as the description above. The co-linearity was determined by comparing physical map and genetic map. The visualization of the map order was carried out using Strudel V. 1.12.03.20 (Bayer et al., 2011), and the heat map showing co-linearity in percentage was produced using MeV V4.9 (Saeed et al., 2003).

### Quantitative trait analysis and visualization

QTL analysis was conducted for all 4-year disease ratings together with the genotyping data and genetic map information. The QTL analysis was conducted using software Windows QTLCartographer version 2.5 (Wang et al., 2011). QTLCartographer uses an active algorithm which considers QTL-environment interactions, various gene actions (additive and dominance), and close linkage. The composite interval mapping (CIM) analysis was conducted by scanning intervals of 1.0 cM between markers and putative QTLs with a window size of 10.0 cM and using the parameters of model 6 and 500 times of permutation with 0.05 significance level along with the function of “Locate QTLs” option to locate QTLs.

## Results

### Improved genetic linkage map

The early version of genetic map for the T-population had 239 mapped loci (Qin et al., 2012), which was improved to 378 marker loci (Pandey et al., 2014). In this study, an additional 45 marker loci were found polymorphic and 40 marker loci were successfully integrated into the existing genetic map. The current improved genetic linkage map has 418 marker loci distributed on 20 linkage groups (LGs) spanning a total genetic map length of 1935.4 cM with map density of 5.3 cM per loci (Table 1, Figure S1). The mapped marker loci per LG varied from 6 (B09) to 42 (A04) with an average of 20.9 loci per LG. Of the 418 mapped markers, 250 marker loci were mapped onto the A sub-genome with a total map distance of 1041.6 cM and a map density of 4.45 cM per loci, while 168 marker loci were mapped onto the 10 LGs of B sub-genome with a total map distance of 893.8 cM and a map density of 6.25 cM per loci (Table 1).

**Table 1:** **Features of the saturated genetic map with 418 mapped loci for the T-population**.

**Linkage group**	**Mapped loci**	**Length of LG (cM)**	**Map density (cM/loci)**
**A SUB-GENOME**			
A01	18	133.4	7.4
A02	11	96.0	8.7
A03	36	176.0	4.9
A04	42	127.2	3.0
A05	24	56.6	2.4
A06	30	151.9	5.1
A07	15	50.0	3.3
A08	36	143.4	4.0
A09	19	66.1	3.5
A10	19	41.0	2.2
Total	250	1041.6	4.45
**B SUB-GENOME**			
B01	18	92.2	5.1
B02	23	68.2	3.0
B03	7	81.2	11.6
B04	21	244.3	11.6
B05	12	50.7	4.2
B06	15	59.4	4.0
B07	22	100.4	4.6
B08	27	50.4	1.9
B09	6	73.2	12.2
B10	17	73.8	4.3
Total	168	893.8	6.25
Grand total	418	1935.4	5.3

### Comparison between the genetic map and the physical map

**Figure 1:**
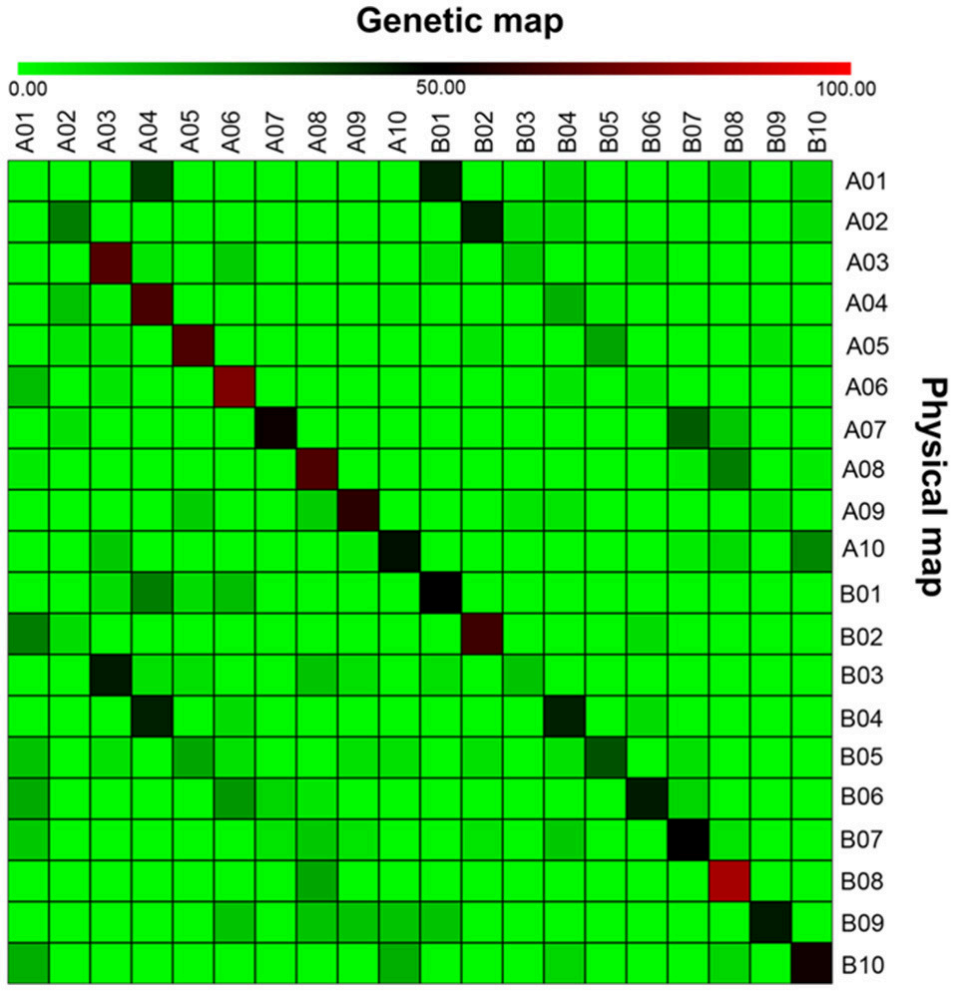
**Genome-wide comparison of mapped markers on the T-population genetic map with the physical map of sub-genomes of the two diploid ancestors of cultivated peanut**. The physical map of the pseudomolecules A01–A10 are from *Arachis duranensis* while the pseudomolecules B01–B10 are from *A. ipaensis*. The linkage groups A01–A10 of genetic map are that from *A. duranensis* while that from B01–B10 of *A. ipaensis*. Gradient color denotes percent similarity between the marker locations in genetic map compared with that of the physical map. The light green color denotes 0% similarity, i.e., no marker in common between that linkage groups and the corresponding pseudomolecules, while red color denotes 100% similarity, i.e., all the markers on a particular linkage group of genetic map were also present on corresponding pseudomolecules of the physical map.

**Figure 2:**
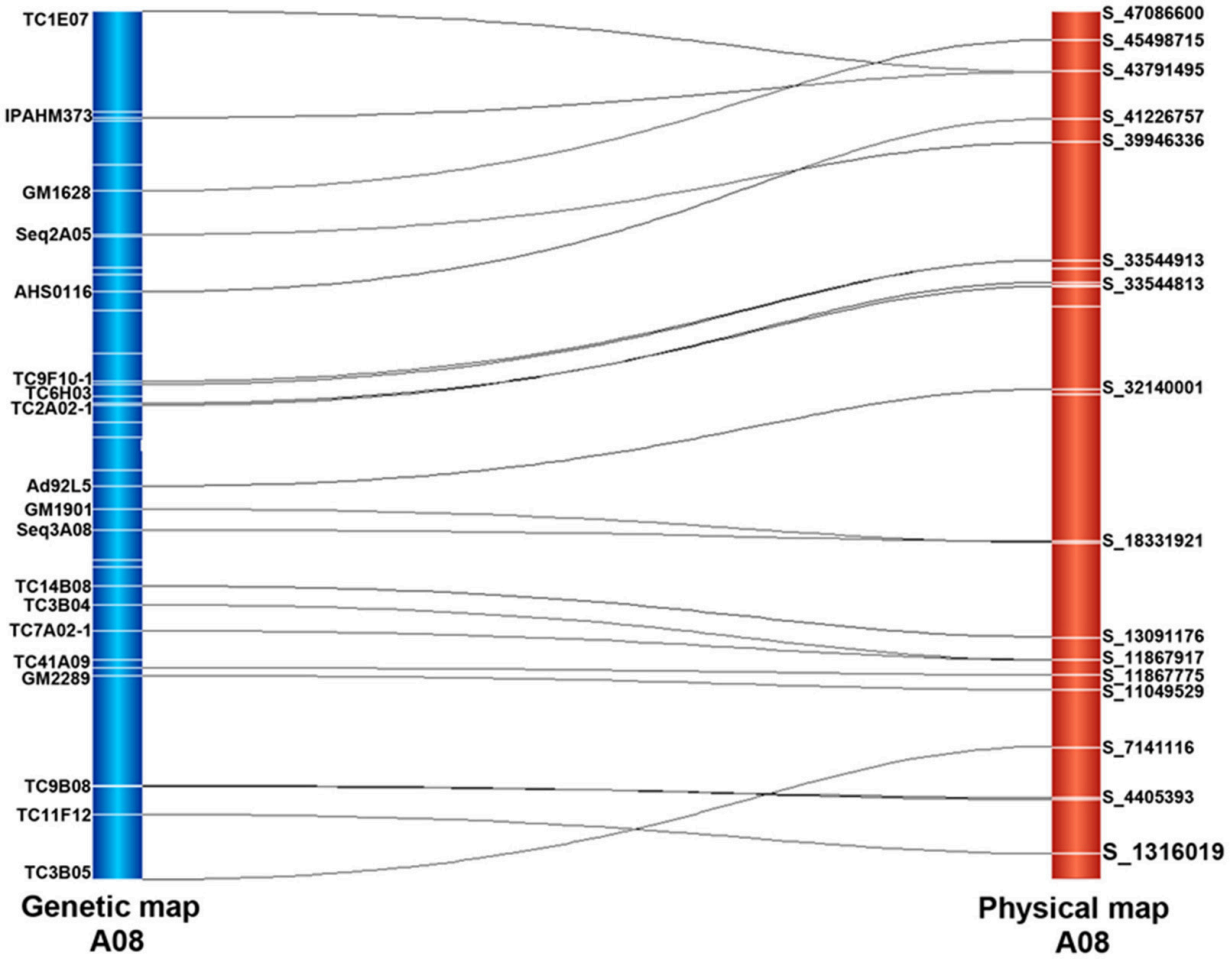
**A representation of co-linearity, the markers mapped on the genetic map A08 of the T-population with the pseudomolecules of the two diploid physical maps**. The lines connecting the two maps indicate that the positions of markers on the genetic map were corresponding to its relative positions on the physical map.

The 403 EST sequences obtained were searched against the peanut reference genome, out of which blast hits were identified for 401 sequences. From the aligned sequences, a total of 59.10% (237) sequences were mapped on A sub-genome while 40.90% (164) sequences were mapped on the B sub-genome (Figure 1). The number of aligned sequences ranged from 16 to 34 on the A sub-genome while the number of mapped sequences ranged from 9 to 25 on the B sub-genome. The maximum 34 and 25 sequences were mapped on “A04” and “B06” pseudomolecules, respectively. The positions of markers on genetic map and physical map were compared to determine the co-linearity between genetic map and physical map. The average co-linearity observed on A sub-genome was 51.73% and that on B sub-genome was 46.35% while overall, 48.36% co-linearity was observed on the entire peanut genome. Individually, markers on pseudomolecules “A10” and “B06” of A and B sub-genomes showed maximum 73.7 and 73.3% co-linearity, while no co-linearity was observed for markers on “A01” pseudomolecule. A representation showing the co-linearity for LG “A08” is given in Figure 2.

### QTLs associated with disease resistance traits

**Figure 3:**
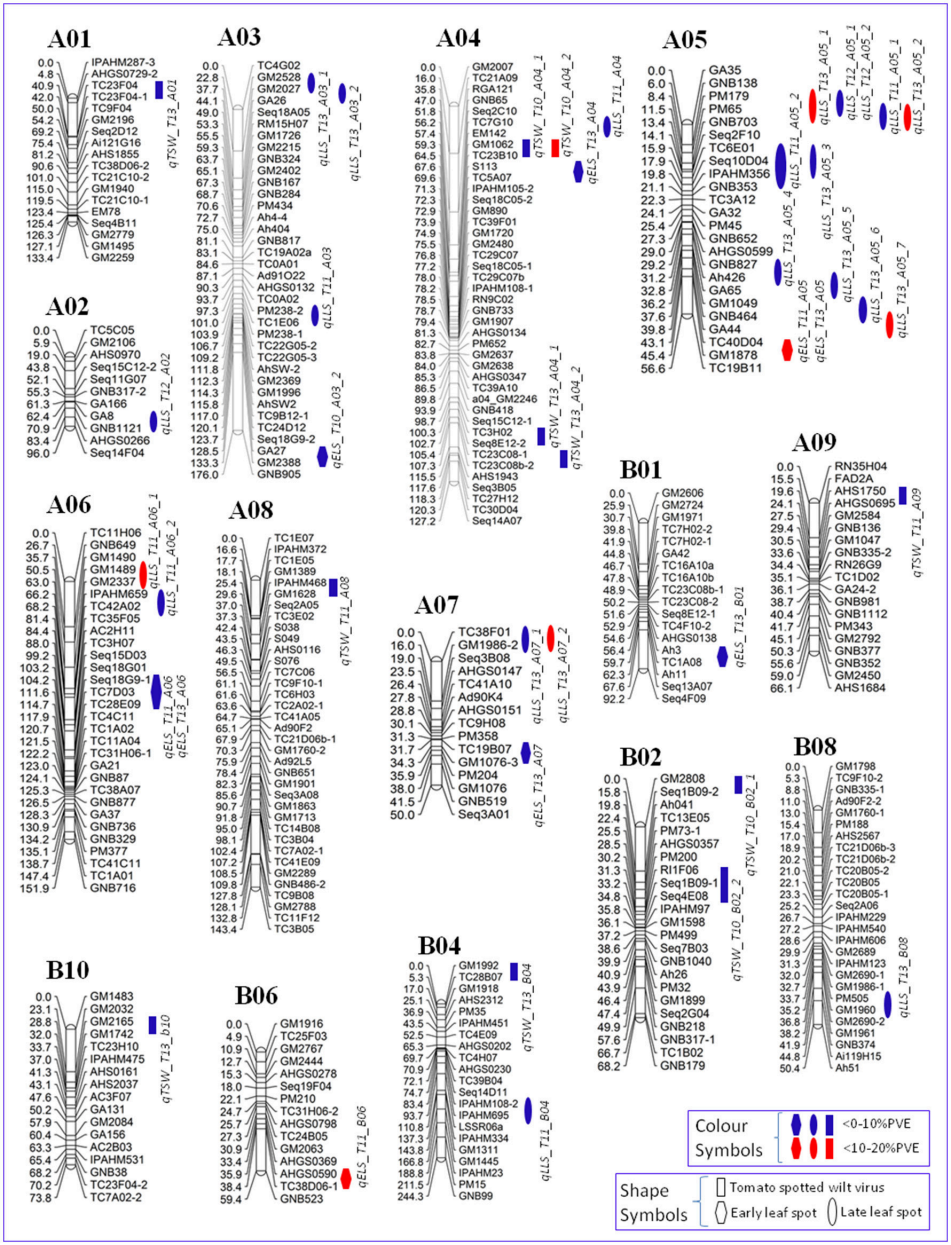
**Genetic map of the T-population from the cross Tifrunner and GT-C20 showing genomic regions with QTLs for resistance to early leaf spot (ELS), late leaf spot (LLS), and Tomato spotted wilt virus (TSWV)**. This figure also shows clusters containing QTL for multiple disease resistance traits.

The QTL analysis using multi-season phenotypic and genotypic data resulted in identification of 42 QTLs for three diseases in the T-population (Tables 2, 3, Figure 3, Table S1). These QTLs were mapped onto 16 LGs (Figure 3) with percentage of phenotypic variation explained (PVE%) ranging from 6.26% (*qELS_T13_A04*) to the maximum of 15.55% (*qLLS_T13_A05_7*) (Table 3). Of the total 42 QTLs, 12 QTLs exhibited >10% PVE and were termed major effect QTLs (Table 3). Furthermore, the distribution of all 42 QTLs across 16 LGs revealed that 34 QTLs were distributed throughout nine LGs of the A sub-genome while eight QTLs were mapped across seven LGs of the B sub-genome. A maximum of 13 QTLs were identified onto LG “A05” followed by six and four QTLs onto LG “A04” and “A03” respectively in the A sub-genome. A maximum of two QTLs each were identified onto LG “B02” and “B04,” followed by one QTL each on the other four LGs in the B sub-genome.

**Table 2:** **Summary of QTLs identified in the T-population for resistance to Tomato spotted wilt virus, early leaf spot, and late leaf spot**.

**Trait/Year**	**Reading**	**QTLs identified**	**Major QTLs**	**LOD value range**	**Phenotypic variation explained (PVE%)**	**Additive effect (a0) range**
**TOMATO SPOTTED WILT VIRUS**						
2010	July	2	1	4.06–6.36	8.95–14.41	−0.53 to 0.47
2010	August	2	–	3.00–3.02	6.74–6.82	−0.28 to 0.27
2011	July	2	–	3.35–3.51	7.15–9.65	0.24 to 0.31
2013	July	5	–	3.00–3.40	7.35–9.29	−0.28 to 0.26
**EARLY LEAF SPOT**						
2010	July	1	1	3.33	11.50	−0.23
2011	August	3	3	3.33–5.93	10.63–12.61	−0.22 to −0.20
2013	July	5	2	3.19–5.76	6.26–13.20	−0.21 to −0.13
**LATE LEAF SPOT**						
2011	September	7	1	3.24–4.55	6.63–10.12	−0.19 to 0.16
2012	September	3	–	3.04–3.59	7.02–9.16	−0.36 to 0.34
2013	August	8	3	3.04–4.82	6.40–15.55	−0.36 to −0.21
2013	September	4	1	3.55– 5.03	8.05–12.35	−0.39 to −0.32
Total		42	12	3.00–6.36	6.26–15.55	−0.53 to 0.47

**Table 3:** **Details on QTLs identified for TSWV, ELS, and LLS resistance in the T-population**.

**S No**.	**QTL name**	**Year**	**Month of Observation**	**Linkage group**	**Marker interval**	**Left marker Physical position**	**Right marker physical position**	**LOD value**	**Phenotypic variation explained (%)**	**Additive effect (a0)**
**TOMATO SPOTTED WILT VIRUS (TSWV)**										
1	*qTSW_T10_A04_1*	2010	July	A04	GM1062–TC23B15	*Aradu.A04_121088160*	–	6.36	14.41	−0.53
2	*qTSW_T10_A04_2*	2010	August	A04	GM1062–TC23B15	*Aradu.A04_121088160*	–	3.02	6.74	−0.28
3	*qTSW_T10_B02_1*	2010	August	B02	GM2808–Seq1B09-2	*Araip.B02_103261801*	*Aradu.A02_20947839*	3	6.82	0.27
4	*qTSW_T10_B02_2*	2010	July	B02	RI1F06–Seq4E08	*Aradu.A02_77240043*	*Araip.B02_64583082*	4.06	8.95	0.47
5	*qTSW_T11_A08*	2011	July	A08	IPAHM468–GM1628	*Aradu.A09_3145922*	*Aradu.A08_45498715*	3.35	7.15	0.24
6	*qTSW_T11_A09*	2011	July	A09	AHS1750–AHGS0695	*Araip.B07_57472323*	*Aradu.A09_111384841*	3.51	9.65	0.31
7	*qTSW_T13_A01*	2013	July	A01	TC23F04–TC23F04-1	*Araip.B10_96894035*	*Araip.B10_96894035*	3.27	9.29	−0.28
8	*qTSW_T13_A04_1*	2013	July	A04	TC3H02–Seq8E12-2	*Aradu.A01_102382032*	*Aradu.A01_104139693*	3.4	7.59	0.26
9	*qTSW_T13_A04_2*	2013	July	A04	TC23C08-1–TC23C08b-2	*Araip.B01_118165090*	*Araip.B01_118165090*	3.14	8.67	0.27
10	*qTSW_T13_B04*	2013	July	B04	GM1992–TC28B07	*Aradu.A01_88729509*	*Aradu.A04_2024488*	3.17	8.99	−0.27
11	*qTSW_T13_B10*	2013	July	B10	GM2165–GM1742	*Araip.B10_1799837*	*Araip.B10_4784882*	3	7.35	−0.25
**EARLY LEAF SPOT (ELS)**										
12	*qELS_T10_A03_2*	2010	July	A03	GA27–GM2388	*Araip.B03_19993020*	*Araip.B03_22414726*	3.33	11.5	−0.23
13	*qELS_T11_A05*	2011	August	A05	TC40D04–GM1878	*Aradu.A05_86235178*	*Araip.B05_6635016*	4.39	11.23	−0.21
14	*qELS_T11_A06*	2011	August	A06	Seq18G9-1–TC28E09	*Araip.B01_123278331*	*Araip.B06_2116158*	5.93	12.61	−0.22
15	*qELS_T11_B06*	2011	August	B06	AHGS0590–TC38D06-1	*Araip.B04_83644293*	*Araip.B02_3444489*	3.33	10.63	−0.2
16	*qELS_T13_A04*	2013	July	A04	S113–TC5A07	–	*Aradu.A04_11614688*	3.25	6.26	−0.15
17	*qELS_T13_A05*	2013	July	A05	TC40D04–GM1878	*Aradu.A05_86235178*	*Araip.B05_6635016*	4.93	12.71	−0.21
18	*qELS_T13_A06*	2013	July	A06	Seq18G9-1–TC28E09	*Araip.B01_123278331*	*Araip.B06_2116158*	5.76	13.2	−0.21
19	*qELS_T13_A07*	2013	July	A07	TC19B07–GM1076-3	*Aradu.A07_26534041*	*Araip.B06_39385375*	3.19	6.67	−0.13
20	*qELS_T13_B01*	2013	July	B01	Ah3–TC1A08	*Araip.B01_118165092*	*Araip.B01_114116578*	3.35	7.02	−0.13
**LATE LEAF SPOT (LLS)**										
21	*qLLS_T11_A03*	2011	September	A03	PM238-2–TC1E06	*Aradu.A03_22168867*	*Aradu.A03_10132301*	4.08	9.11	−0.16
22	*qLLS_T11_A04*	2011	September	A04	TC7G10–EM142	*Aradu.A04_3479736*	*Aradu.A04_121074477*	3.26	6.63	−0.18
23	*qLLS_T11_A05_1*	2011	September	A05	PM65–GNB703	*Araip.B05_18606218*	*Aradu.A09_93893154*	3.38	8.63	−0.18
24	*qLLS_T11_A05_2*	2011	September	A05	TC6E01–GNB353	*Aradu.A05_33061631*	*Aradu.A05_17795698*	4.55	9.55	−0.19
25	*qLLS_T11_A06_1*	2011	September	A06	GM1489–GM2337	*Aradu.A06_1258634*	*Araip.B06_18837557*	4.32	15.12	−0.19
26	*qLLS_T11_A06_2*	2011	September	A06	IPAHM659–TC42A02	*Aradu.A06_7370126*	*Aradu.A06_2062118*	3.56	9.61	−0.19
27	*qLLS_T11_B04*	2011	September	B04	IPAHM108-2–IPAHM695	*Aradu.A04_111588972*	*Aradu.A04_108179281*	3.24	9.33	0.16
28	*qLLS_T12_A02*	2012	September	A02	GA8–GNB1121	*Aradu.A05_23339934*	*Araip.B02_58499596*	3.04	7.02	0.34
29	*qLLS_T12_A05_1*	2012	September	A05	PM179–PM65	–	*Araip.B05_18606218*	3.59	8.42	−0.36
30	*qLLS_T12_A05_2*	2012	September	A05	PM179–PM65	–	*Araip.B05_18606218*	3.23	9.16	−0.36
31	*qLLS_T13_A03_1*	2013	September	A03	GM2528–GM2027	*Araip.B03_126672393*	*Araip.B03_124049435*	3.7	8.78	−0.33
32	*qLLS_T13_A03_2*	2013	August	A03	GM2027–GA26	*Araip.B03_124049435*	*Aradu.A05_109137871*	3.11	6.4	−0.21
33	*qLLS_T13_A05_1*	2013	September	A05	PM179–GNB703	–	*Aradu.A09_93893154*	5.03	12.35	−0.39
34	*qLLS_T13_A05_2*	2013	August	A05	PM65–GNB703	*Araip.B05_18606218*	*Aradu.A09_93893154*	4.82	15.13	−0.27
35	*qLLS_T13_A05_3*	2013	August	A05	TC6E01 –IPAHM356	*Aradu.A05_33061631*	*Aradu.A05_19076425*	3.8	8.9	−0.26
36	*qLLS_T13_A05_4*	2013	August	A05	GNB827–Ah426	*Aradu.A05_37578655*	–	3.72	9.27	−0.27
37	*qLLS_T13_A05_5*	2013	August	A05	Ah426–GA65	–	*Araip.B05_16912303*	3.61	9.74	−0.3
38	*qLLS_T13_A05_6*	2013	August	A05	GM1049–GNB464	*Aradu.A05_62322817*	*Aradu.A05_85047929*	3.04	7.41	−0.26
39	*qLLS_T13_A05_7*	2013	August	A05	GNB464–GA44	*Aradu.A05_85047929*	*Araip.B03_128254123*	5.26	15.55	−0.36
40	*qLLS_T13_A07_1*	2013	September	A07	TC38F01–GM1986-2	*Aradu.A07_73062983*	*Aradu.A07_53236894*	3.55	8.05	−0.32
41	*qLLS_T13_A07_2*	2013	August	A07	TC38F01–GM1986-2	*Aradu.A07_73062983*	*Aradu.A07_53236894*	3.82	12.82	−0.3
42	*qLLS_T13_B08*	2013	September	B08	PM505–GM1960	*Araip.B07_79877009*	–	3.69	9.15	−0.33

### QTLs associated with resistance to early leaf spot (ELS)

The phenotyping data recorded for ELS during 3 years (2010, 2011, and 2013) at Tifton, GA was used in QTL analysis. A total of nine QTLs were identified for ELS with five in 2013, three in 2011 and one in 2010, with PVE ranging from 6.26% (*qELS_T13_A04*) to 13.20% (*qELS_T13_A06*) (Table 3). All the nine QTLs were distributed over seven LGs i.e., seven QTLs on five LGs of the A sub-genome while two QTLs were on two LGs of the B sub-genome. The LG “A05” and “A06” harbored two QTLs each, *qELS_T11_A05*, and *qELS_T13_A05* on “A05,” and *qELS_T11_A06* and *qELS_T13_A06* on “A06.” For the remaining five QTLs, single QTL was mapped on each LG, “A03,” “A04,” “A07,” “B01,” and “B06.”

If the QTL for a particular trait was identified on the same genomic region for two or more than two times (environments), it was defined as “Consistent QTL.” For example, two QTLs namely *qELS_T11_A05* (August, 2011) and *qELS_T13_A05* (July, 2013) were found on LG “A05” in genomic region (TC40D04–GM1878). Similarly, two QTLs namely *qELS_T11_A06* (August, 2011) and *qELS_T13_A06* (July, 2013) were found on LG “A06” in the genomic region (Seq18G9-1–TC28E09). All nine QTLs associated with resistance to ELS were contributed by the parent Tifrunner, and six QTLs had major effect with more than 10% PVE ranging from 10.63% (*qELS_T11_B06*) to 13.20% (*qELS_T13_A06*). Interestingly, both LG “A06” and “B06” had two major QTLs.

### QTLs associated with resistance to late leaf spot (LLS)

Disease scoring for late leaf spot (LLS) was observed in 3 years (2011, 2012, and 2013). A total of 22 QTLs were identified with PVE ranging from 6.40% (*qLLS_T13_A03_2*) to 15.55% (*qLLS_T13_A05_7*) (Table 3), including 12 QTLs identified for the year 2013 followed by seven in 2011 and three in 2012. Of the 22 QTLs, five QTLs had major effects with PVE ranging from 10.12 to 15.55%, in which LG “A05” possessed three major QTLs with PVE as high as 15.55% (*qLLS_T13_A05_7*). Interestingly, 11 QTLs were mapped on LG “A05” across all 3 years (Table 3). In addition, “A05” had one consistent QTL in the genomic region between marker PM65–GNB703 for August, 2013 (*qLLS_T13_A05_2)* and September, 2011 (*qLLS_T11_A05_1*). All the 11 QTLs associated with resistance to LLS on LG “A05” were contributed from the parent “Tifrunner.” Overall, 20 QTLs were contributed by the resistant parent “Tifrunner” in contrast to two QTLs, at the genomic regions of IPAHM108-2–IPAHM695 and GA8–GNB1121, which were contributed from the susceptible parent “GT-C20” (Table 3).

### QTLs associated with resistance to tomato spotted wilt virus (TSWV)

Field evaluations were conducted for TSWV disease ratings for 3 years (2010, 2011, and 2013), and 11 QTLs were identified with PVE ranging from 6.74% (*qTSW_T10_A04_2*) to 14.41% (*qTSW_T10_A04_1*) (Table 3). In 2010, four QTLs were detected, two each on LG “A04” and “B02.” Interestingly two of these QTLs namely *qTSW_T10_A04_1* and *qTSW_T10_A04_2* shared the same genomic region located between GM1062–TC23B10. The other two QTLs were detected on the same LG “B02” but at different positions. These two QTLs on LG “A04” were contributed by the parent “Tifrunner” and the other two QTLs on “B02” were contributed by parent “GT-C20.” In 2011, only two QTLs were found, one on “A08” and the other on “A09” and both contributed by the parent “GT-C20.” In the year 2013, five QTLs were mapped on four different LGs. Two QTLs namely *qTSW_T13_A04_1* and *qTSW_T13_A04_2* were mapped on LG “A04,” while the other three QTLs (*qTSW_T13_A01, qTSW_T13_B04*, and *qTSW_T13_B10*) were located on LG “A01,” “B04,” and “B10,” respectively. There was only one major QTL with PVE more than 10% (14.41%, *qTSW_T10_A04_1*).

## Discussion

Peanut, also called groundnut, is the second most important legume oilseed crop in the world. Genetic improvement of yield and production is always the ultimate goal. Peanut is susceptible to many diseases and the cost of disease control can be high. Since MAS and genomics-assisted breeding (GAB) have several advantages over conventional breeding approaches, it is advisable to identify linked markers and then use these markers in improving the target traits through GAB. Therefore, we have used a RIL population called T-population for phenotyping and genotyping followed by construction of an improved genetic map and identification of QTLs associated with three important diseases. This study included 4 years of field evaluations of disease severity for early leaf spot (ELS), late leaf spot (LLS) and TSWV from 2010 to 2013, and each year there were two planting dates (total eight field trials) in order to obtain better field rating (all naturally occurring). The genetic linkage map was also improved to 418 marker loci (Table 1, Figure S1). Major QTLs with larger contribution to the phenotypic variation were identified for all three diseases (Table 2). This study showed good co-linearity between the genetic map and the physical map (Figures 1, 2). These QTLs will be further studied for fine mapping of linked markers and identification of potential candidate genes by high density SNP array (Pandey et al., 2017) and whole genome resequencing (WGRS) (Agarwal et al., 2017) which could be deployed for marker-assisted selection (MAS). The validation and deployment of linked markers, flanking these QTLs would further facilitate candidate resistance genes discovery and also to genetically improve peanut cultivars with enhanced disease resistance for TSWV and leaf spots through GAB.

### Significance of trait phenotyping for precise QTL identification

Phenotyping is considered as the foundation for identifying marker-trait associations; hence precise phenotyping is needed to avoid false detection of QTLs and markers. It has been observed that the majority of the studies have used phenotypic data on disease screening in controlled environments such as greenhouse trials with manual inoculation of disease. The limitation is rating the true performance of plant response to disease pathogen in a controlled environment because the environmental effect does play an important role in disease development. The other limitation is the limited sample number. Therefore, use of natural field screenings for disease reactions is preferable to generate representative phenotypic data. Often, the QTLs obtained through controlled environment like greenhouse screening contribute higher PVE, but these QTLs exhibit very low PVE in field conditions. On the other hand, the QTLs identified in natural fields are often consistent in repeat field trails, but it is very labor intensive and costly.

In the current study, screening was done for three diseases of peanut under field conditions for 4 years i.e., 2010–2013. To insure better disease infection, there were two planting dates every year (April and May). The first planting was done in April in order to improve chance for TSWV, while the second planting was done in May in order to reduce the interference of TSWV to leaf spots (ELS and LLS) disease ratings, and increase the chance of leaf spot epidemics. Moreover, disease was scored more than once during each cropping season. It was observed that infection was more for ELS in the year 2010, while moderate ELS infection was observed in the year 2011 and 2013. High LLS infection was noted in the year 2011 and 2013. High TSWV infection was observed in 2010 and 2013.

### Improved genetic map density and its co-linearity with diploid ancestor genomes

A genetic map with high marker density is essential for effective QTL identification. In order to improve the density of our genetic map, we screened more markers and added 40 new markers to the genetic map with 418 marker loci distributed onto 20 LGs covering a total map distance of 1935.4 cM with a map density of 5.3 cM per loci. The previous version of the genetic map (Pandey et al., 2014) had 378 marker loci with map density of 7.0 cM per loci. The current map has a higher map density than the previous version, and the A sub-genome had higher number of loci (231) than the B sub-genome (151) indicating that the A sub-genome is more diverse than the B sub-genome (Bertioli et al., 2016).

In the last decade, the international peanut community has progressed from a complete lack of molecular markers to the release of the genome sequences for the two diploid progenitor species (Guo et al., 2016). Various genetic maps based on RIL populations have been developed (Pandey et al., 2012b, 2016). The first SSR-based genetic map was constructed in 2009 with 135 marker loci using the RIL population developed from the cross TAG 24 × ICGV 86031 (Varshney et al., 2009). Although, that map had less number of loci, hence the emphasis had been given to integrate more marker loci by taking initial map as a base, and the outcome was a map with 191 marker loci with the same RIL population (Ravi et al., 2011). Similarly, two other genetic maps developed through the cross of TAG 24 × GPBD 4 and TG 26 × GPBD 4 had 56 and 45 marker loci, respectively (Khedikar et al., 2010; Sarvamangala et al., 2011). Later Sujay et al. (2012) improved both these genetic maps with 188 and 181 marker loci, respectively. Similarly, the first version of genetic maps for the S-population and T-population were prepared with 172 and 236 marker loci, respectively (Qin et al., 2012), which were later improved to maps with 206 and 378 marker loci, respectively (Pandey et al., 2014). Now, these two maps are further saturated leading to the third version with 248 (Khera et al., 2016) and 418 marker loci, respectively.

Genome sequences for the diploid peanut progenitors are available (Bertioli et al., 2016; Chen et al., 2016), which provides substantial opportunities for different genomics studies, including comparative genomics (Pandey et al., 2016). Prediction of the physical location of EST-SSR markers on the two diploid genome assemblies helped in getting insights on the co-linearity between the genetic map and the two sub-genome physical maps. Despite fair co-linearity observed across two sub-genomes, it was interesting to observe that few marker loci with assigned physical locations on one chromosome of a sub-genome were also mapped on the respective homeologous chromosomes of another sub-genome, suggesting duplication of sequences in the homeologous chromosomes in peanut. These findings will facilitate the determination of the QTLs' physical locations, identification of functional resistance candidate genes and marker development.

### Clusters harboring multiple resistance QTLs identified on LG “A05”

The main objective of this study was to identify QTLs linked to disease resistance. Use of an improved genetic map together with multi-year field data resulted in identification of 42 QTLs, with 12 QTLs exhibiting major effects, i.e., more than 10% PVE. Further, more QTLs (34 QTLs) were mapped on the A sub-genome as compared to only eight QTLs mapped on the B sub-genome. The above trend was seen in all three disease resistance traits. For example, of the 11 QTLs identified for TSWV, there were seven QTLs mapped on the A sub-genome and four QTLs located on the B sub-genome. Similarly, of the nine QTLs identified for ELS, seven QTLs were mapped on the A sub-genome while two QTLs mapped on the B sub-genome. As for LLS, 20 of the 22 QTLs mapped on the A sub-genome and two QTLs mapped on the B sub-genome. These results indicate that the A sub-genome is sheltering more resistance genes than the B sub-genome, which is in agreement with Bertioli et al. (2016), who reported that there are more nucleotide-binding–leucine-rich repeat (NB-LRR)-encoding disease resistance-like genes in the “A” genome than in the “B” genome (397 and 345 of these genes in the *A. duranensis* and *A. ipaensis* genotypes, respectively).

A single LG “A05” harbored 13 QTLs i.e., including 11 QTLs for LLS and two for ELS. These 13 QTLs clustered on three genomic regions of LG “A05,” upper arm, lower arm and the central region, respectively (Figure 3). The upper arm of “A05” has five QTLs with PVE up to 12.35%, around marker “PM65,” for LLS across 3 years. The central region of “A05” has six QTLs for LLS, and it may have two sub-clusters, two QTLs at around marker “IPAHMM356” with PVE up to 9.55% and three QTLs around markers “Ah426” and “GNB464” with PVE up to 15.55%. Interestingly, the low arm of “A05” has two QTLs for ELS between markers “TC40D04” and “GM1878” with PVE up to 12.71% across 2 years phenotypic data (Figure 3). Recently, Khera et al. (2016) reported two QTLs mapped on LG “A05,” one for ELS around marker “PM65” and one for LLS at marker “GM1878,” both also mapped on LG “A05” of this study. Comparisons showed high similarity between these two LGs named “A05” in this study and Khera et al. (2016), which were developed from different mapping populations. There were 11 markers of LG “A05” in this study mapped on the LG “A05” of Khera et al. (2016), but the two QTLs of Khera et al. (2016) were at the same genomic regions but for different phenotypic traits. The most likely explanation is that the phenotypic evaluation were conducted in the field with all natural occurring inoculations and the visual disease rating will include both diseases, ELS and LLS. Therefore, these QTLs harbored on these LG regions provide possible good disease resistance and warrant further investigation for validation and possible use in assistance to breeding selection.

In summary, the present study provided an improved genetic map with more markers and showed co-linearity of the genetic map with two diploid progenitor physical maps. A total of 42 QTLs associated with disease resistances, 34 were mapped on the A sub-genome while only eight mapped on the B sub-genome. This suggests that the A sub-genome chromosomes have more resistance genes than the B sub-genome. The identified genomic regions controlling leaf spots and TSWV resistance and the linked markers will be further studied and these genomic regions will be validated for possible application in molecular breeding for developing peanut varieties with improved disease resistance.

## Author contributions

Conceived and designed the experiments: BG and RV. Performed the experiments: MP, HW, and PK. Analyzed data: PK, MP, HW, MV, SK, and XW. Field evaluation: BG, AC, and CH. Wrote the paper: PK, MP, HW, MV, XW, RV, and BG.

### Conflict of interest statement

The authors declare that the research was conducted in the absence of any commercial or financial relationships that could be construed as a potential conflict of interest.

## Supplementary material

Figure S1 **Genetic linkage map of the T-population from the cross Tifrunner and GT-C20**. This genetic map shows map location and order of 418 mapped loci on the 20 linkage groups (A01–A10, B01–B10).

Table S1 **List of QTLs identified in the T-population for the three disease resistance traits (early leaf spot, late leaf spot, and Tomato spotted wilt virus)**.
