# Supplementary figures and images for: Genetic Dissection of Novel QTLs for Resistance to Leaf Spots and Tomato Spotted Wilt Virus in Peanut (Arachis hypogaea L.)
Source: Front Plant Sci. 2017 Jan 31;8:25. doi: 10.3389/fpls.2017.00025 (PMC5281592; doi:10.3389/fpls.2017.00025)

## Slide 1
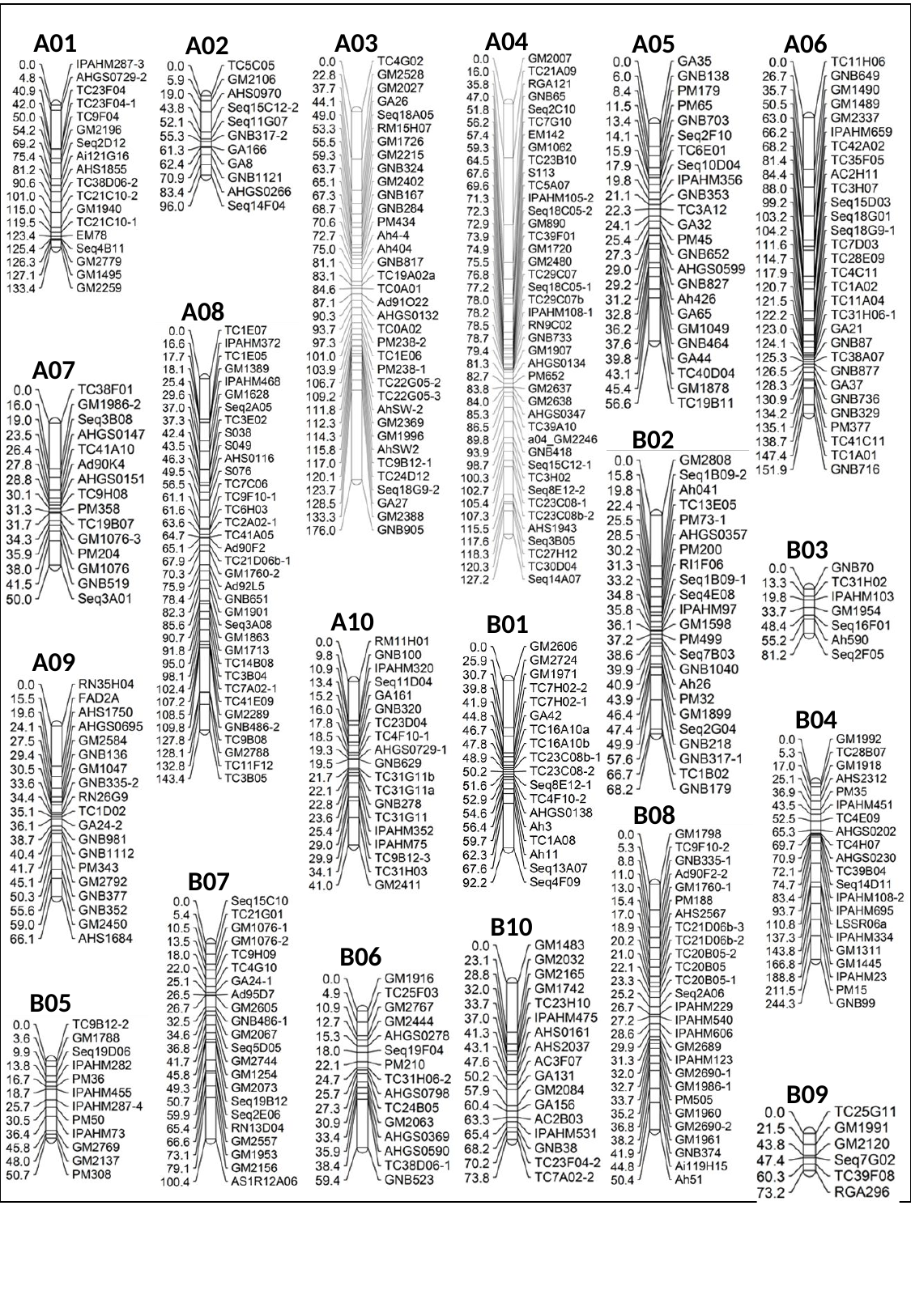

A04
A01
A03
A05
A06
A02
A08
A07
B02
B03
A10
B01
A09
B04
B08
B07
B10
B06
B05
B09

Supplement: Figure S1 — Genetic linkage map of the T-population from the cross Tifrunner and GT-C20. This genetic map shows map location and order of 418 mapped loci on the 20 linkage groups (A01–A10, B01–B10). [file Presentation1.PPTX]
